# DNA repair genes *XRCC1* and *XRCC3* polymorphisms and their relationship with the level of micronuclei in breast cancer patients

**DOI:** 10.1590/S1415-47572010005000082

**Published:** 2010-12-01

**Authors:** Raquel A. Santos, Ana Claudia Teixeira, Monica B. Mayorano, Helio H. A. Carrara, Jurandyr M. Andrade, Catarina S. Takahashi

**Affiliations:** 1 Departamento de Genética, Faculdade de Medicina de Ribeirão Preto, Universidade de São Paulo, Ribeirão Preto, SPBrazil; 2 Departamento de Ginecologia e Obstetrícia, Faculdade de Medicina de Ribeirão Preto, Universidade de São Paulo, Ribeirão Preto, SPBrazil; 3 Departamento de Biologia, Faculdade de Filosofia, Ciências e Letras de Ribeirão Preto, Universidade de São Paulo, Ribeirão Preto, SPBrazil

**Keywords:** DNA repair polymorphisms, breast cancer, micronucleus assay

## Abstract

Breast cancer (BC) is the most prevalent type worldwide, besides being one of the most common causes of death among women. It has been suggested that sporadic BC is most likely caused by low-penetrance genes, including those involved in DNA repair mechanisms. Furthermore, the accumulation of DNA damage may contribute to breast carcinogenesis. In the present study, the relationship between two DNA repair genes, viz., *XRCC1* (*Arg399Gln*) and *XRCC3* (*Thr241Met*) polymorphisms, and the levels of chromosome damage detected in 65 untreated BC women and 85 healthy controls, was investigated. Chromosome damage was evaluated through micronucleus assaying, and genotypes determined by PCR-RFLP methodology. The results showed no alteration in the risk of BC and DNA damage brought about by either *XRCC1* (*Arg399Gln*) or *XRCC3* (*Thr241Met*) action in either of the two groups. Nevertheless, on evaluating BC risk in women presenting levels of chromosome damage above the mean, the *XRCC3Thr241Met* polymorphism was found to be more frequent in the BC group than in the control, thereby leading to the conclusion that there is a slight association between *XRCC3* (241 C/T) genotypes and BC risk in the subgroups with higher levels of chromosome damage.

Breast cancer (BC) is one of the most common causes of death among women, with every indication of a slow and steady decrease in the age of onset. The risk factors for BC include the early age of menarche, delayed menopause, the use of contraceptives, hormonal replacement therapy, the above average body-mass index, exposure to environmental pollutants, smoking and alcohol use (Kristensen and Borresen-Dale, 2000; Hulka and Moorman, 2001; Kang *et al.*, 2002). However, it is generally believed that the initiation of BC is a consequence of cumulative genetic damage thereby leading to genetic alterations, with subsequent activation of proto-oncogenes and inactivation of tumor-suppressor genes (Mitrunen and Hirvonen, 2003). A large number of genetic variants associated with BC risk have been identified in genes involved in a wide variety of functions, including steroid hormone metabolism, detoxification of environmental carcinogens, tumor suppression and DNA damage repair (Dunning *et al.*, 1999).

Polymorphisms in DNA repair genes are common. Studies have revealed that the effects of these polymorphisms on DNA repair ability contribute to individual differences (Pachkowski *et al.*, 2006). There are two important genes involved in this process. One, the X-Ray Repair Cross Complementing 1 (*XRCC1*) gene involved in the Base Excision Repair (BER) pathway, is linked with a scaffolding protein that directly associates with other proteins, such as DNA polymerase ß , PARP (ADP-ribose polymerase) and DNA ligase III, in a complex that facilitates processes of BER DNA repair (Caldecott *et al.*, 2003). The other, *XRCC3*, one of the key components of the homologous repair (HR) pathway, functions in the cross-link repair of DNA double-strand breaks (DSBs) by interacting and stabilizing Rad51 (Schild *et al.*, 2000; Thompson and Schild, 2002).

It is common knowledge that chromosome damage results from non- or misrepaired DSBs, with many polymorphisms, such as those of DNA repair genes, having been associated with increased cancer risk, and a possibly even higher level of chromosome damage (Norppa 2004).

Thus, the aim hereby was to investigate the relationship between *XRCC1* (*Arg399Gln*) and *XRCC3* (*Thr241Met*) SNPs and chromosomal damage in untreated BC women and healthy controls.

Blood samples for genotyping and micronucleus assaying were obtained from 65 untreated women, with ages ranging from 25 to 60 (mean age 50.6), diagnosed with *in situ* or invasive ductal breast carcinoma and free from any pathological manifestation associated with the use of medication and possibly leading to DNA damage. Of the 65 women in the patient group 33 (50.7%) were post- and 32 (49.3%) pre-menopausal. The control group consisted of 85 women, ages ranging from 25 to 60 (mean age 48.7) with 42 (49.4%) post- and 43 (50.6%) premenopausal. Each was enrolled in the study after detailed investigation, thereby ensuring the absence from any form of breast pathology. These were matched to patients according to the following variables: all the volunteers came from the same geographical location, with identical dietary habits, and without prior occupational exposure to genotoxic chemicals. None reported alcohol consumption, genotoxic medicine intake, the presence of known inherited genetic disorders or chronic diseases, or the exposure to ionizing or non-ionizing radiation, even for diagnostic or therapeutic purposes, for at least one month previously. The investigation received prior approval by the National Ethics Committee (CONEP: 1217/2004), and was undertaken in accordance with defined ethical standards. Informed consent was obtained from patients and controls before inclusion in the study and sample collection.

Genomic DNA samples were obtained from blood lymphocytes for genotyping by using a Wizard® Genomic DNA Purification Kit (Promega, Madison, WI). G399A polymorphism of the *XRCC1* gene was determined by PCR-RFLP with the following primers: sense, 5'-TCTCCCTTGGTCTCCAACCT-3' and antisense, 5'-AGTAGTCTGCTGGCTCTGG-3'. The 402 bp product was digested overnight with 5 U of the restriction enzyme *MspI*. The G allele was digested into 269 and 133 bp fragments. Nevertheless, when the A allele was present, the 402 bp fragment remained intact. C241T polymorphism of the *XRCC3* gene was genotyped with the following primers: sense, 5'-GGTCGAGTGACAGTCCAAAC-3' and antisense, 5'-TGCAACGGCTGAGGGTCTT-3'. The 455 bp product was digested overnight with 5 U of the restriction enzyme *Nla*III. The Leu allele was digested into 210, 140 and 105 bp fragments.

In order to stimulate cell proliferation, lymphocyte cultures were prepared for micronucleus assaying by combining 0.5 mL of isolated lymphocytes in plasma with 5 mL of a complete medium containing 78% of RPMI (Sigma-Aldrich Co., USA), 20% inactivated fetal bovine serum (Gibco-Invitrogen, Denmark), and both of the antibiotics penicillin (5 μg/mL, Sigma-Aldrich Co., USA) and streptomycin (10 μg/mL, Sigma-Aldrich Co., USA), as well as 2% phytohemagglutinin (Life Technologies, Grand Island, NY, USA). Cultures were incubated at 37 °C. According to the Fenech and Morley (1985) method, after 44 h of incubation, cytochalasin B (Sigma-Aldrich Co., USA) was added to the cultures to a final concentration of 4 μg/mL. The cultures were stopped after 72 h, whereat the cells were harvested by centrifugation, submitted to cold hypotonic treatment (1% of sodium citrate), and fixed in two changes of methanol-acetic acid (3:1). The fixed cells were spread onto pre-cleaned glass slides, air-dried, and then stained with a Giemsa solution (Sörensen Buffer, pH 6.8) for 7 min. 1000 binucleated cells were analyzed, whereupon micronucleus frequency (MNF), micronucleated cell frequency (MCF) and the nuclear division index (NDI) were determined for each cell according to criteria described by Fenech (2000).

The Mann-Whitney statistical test was applied for comparing MNF between patients and controls. The level of DNA damage in different genotypes was analyzed with one-way ANOVA, whereas statistical differences between groups for BC risk was calculated using Fisher's exact test (two-tailed). On considering that high chromosome damage could possibly be associated with inefficient DNA repair, subjects with MN frequencies higher than the mean for the respective group, were selected for further analysis, as recommended by Synowiec *et al.* (2008). Crude odds ratios (ORs) were calculated and obtained with 95% confidence intervals (CIs). Results were considered significant when p < 0.05.

The polymorphic variants of the two DNA repair genes, *XRCC1* (399G/A) and *XRCC3* (241 C/T), and genotype distribution of the BC and control groups, were in agreement with those predicted by Hardy-Weinberg equilibrium. There was no difference between patients and controls, as regards *XRCC1* and *XRCC3* genotype frequencies (Table 1). Although it has been suggested that *XRCC1* and *XRCC3* polymorphisms are involved in BC risk, we did not find such correlation, mainly due to the small sample size. Nevertheless, it has been revealed that subtle defects in DNA repair capacity, arising from low-penetrance genes or their combinations, are modified by other genetically determined or environmental risk factors and are correlated with BC risk (Synowiec *et al.*, 2008).

We are aware that this is not an epidemiological study. Rather, our primary objective was to evaluate the correlation between genotype (variants of DNA repair genes) and phenotype (spontaneous chromosome damage). The results obtained after genotype analysis and chromosome damage expressed by MNF are presented in Table 1. In both patient and control groups, no genetic variant of *XRCC1* or *XRCC3* influenced the frequency of micronuclei detected in peripheral lymphocytes. We have previously reported that untreated Brazilian BC patients displayed higher levels of chromosome damage than healthy controls (Santos *et al.*, 2010). Furthermore, it has been suggested that genome damage in lymphocytes may be correlated with cancer-initiating events in target tissues, via a common genetic, dietary or environmental factor (Bonassi *et al.*, 2007). Hence, genetic polymorphisms might explain part of the association between chromosome damage levels and cancer risk (Norppa, 2004). However, in a pooled analysis, Mateuca *et al.* (2008) suggested that single DNA repair gene polymorphisms are not likely to have a major impact on MN frequencies, whereas combinations of different DNA repair genes, and the interplay between *hOGG1*^*326*^, *XRCC1*^*399*^, *XRCC3*^*241*^, genotypes and environmental factors are more likely to modulate MN levels.

Table 2 shows the distribution of *XRCC1* (399G/A) and *XRCC3* (241 C/T) genotypes in groups of patients and controls with higher than mean level of spontaneous DNA damage in both (19.3 for patients and 10.4 for controls). On considering *XRCC1* gene variants, no correlation between the frequencies of genotypes and MN was found. On the other hand, *XRCC3* polymorphism was only slightly associated with BC risk in those individuals that presented higher levels of chromosome damage. This was interesting, especially when considering that susceptibility and risk biomarkers contribute to identifying high-risk subgroups of the population, independent of their association with exposure or involvement in a defined pathway or mechanism (Boffeta, 2009). Notwithstanding, such a correlation cannot be emphasized, especially on considering that when lymphocytes of BC patients and healthy controls were challenged to repair the *in vitro* etoposide-induced DNA damage, the response was similar in both groups, as previously demonstrated (Teixeira *et al.*, 2009). Moreover, the manifestation of the effects of DNA repair enzyme polymorphisms may be quite different between breast tissue undergoing continual exposure to the effects of hormones and lymphocytes.

In conclusion, the levels of chromosome damage observed in breast cancer patients and healthy controls were not associated to the *XRCC1* (399G/A) and *XRCC3* (241 C/T) genotypes. Nevertheless there actually was a weak association between several other *XRCC3* genotypes and BC risk in the subgroup where this damage was higher. Such an association must be interpreted with caution, when considering that the odds ratios obtained, even though pertaining to low-penetrance genes, were statistically significant. Thus, only the common expression of a considerable number of such genes could possibly change this statistical significance into a biological or medical one.

## Figures and Tables

**Table 1:** The genotype frequencies of *XRCC1* and *XRCC3* gene variants, and levels of chromosome damage as evaluated by micronucleus (MN) assay in untreated breast cancer patients and controls.

Genotype	Breast cancer patients (n = 65)			Controls (n = 85)		OR (95% CI)	Breast cancer patients MNF (‰) M ± SD	Controls MNF (‰) M ± SD
	N	Frequency		N	Frequency			
*XRCC1*								
Arg/Arg	24	0.37		24	0.28	1.0 (reference)	16.9 ± 10	10.1 ± 5.8
Arg/Gln	39	0.60		53	0.62	0.73 (0.3-1.4)	20.1 ± 11.1	8.9 ± 6.5
Gln/Gln	2	0.03		8	0.10	0.25 (0.04-1.3)	33 ± 5.6	10.8 ± 5.3
Arg/Gln+Gln/Gln	41	0.63		61	0.72	0.6 (0.3-1.3)	20.7 ± 10.8	9.3 ± 6.2
							p = 0.2	p = 0.7

*XRCC3*								
Thr/Thr	28	0.43		49	0.58	1.0 (reference)	18.7 ± 8.9	11.2 ± 6.3
Thr/Met	31	0.48		29	0.34	1.8 (0.9-3.7)	19.7 ± 12.2	8.3 ± 5.3
Met/Met	6	0.09		7	0.08	1.5 (0.4-4.9)	20.3 ± 12.2	10.8 ± 7.8
Thr/Met+Met/Met	37	0.57		36	0.42	1.7 (0.9-3.4)	19.8 ± 12.1	9.3 ± 6
							p = 0.9	p = 0.3

MNF: micronucleus frequency; OR: odds ratio; M: mean; SD: standard deviation.

**Table 2:** DNA repair-gene polymorphisms in breast cancer patients and controls with high levels of chromosome damage.

Genotype	Breast cancer patients (n = 32)			Controls (n = 21)		OR (95% CI)
	Number	Frequency		Number	Frequency	
*XRCC1*						
Arg/Arg	9	0.28		4	0.19	1.0 (reference)
Arg/Gln+Gln/Gln	23	0.72		17	0.81	1.6 (0.4-6.3)

*XRCC3*						
Thr/Thr	12	0.37		15	0.71	1.0 (reference)
Thr/Met+Met/Met	20	0.63		6	0.29	4.1 (1.2-13.6)↑

OR: odds ratio.
